# Defensive peripersonal space is modified by a learnt protective posture

**DOI:** 10.1038/s41598-019-43258-8

**Published:** 2019-05-01

**Authors:** Monica Biggio, Ambra Bisio, Piero Ruggeri, Marco Bove

**Affiliations:** Department of Experimental Medicine, Section of Human Physiology and Centro Polifunzionale di Scienze Motorie, Viale Benedetto XV 3, 16132 Genoa, Italy

**Keywords:** Cognitive neuroscience, Reflexes

## Abstract

The Hand Blink Reflex (HBR) is a subcortical defensive response, elicited by the electrical stimulation of the median nerve. HBR increases when the stimulated hand is inside the defensive peripersonalspace (DPPS) of the face. However, the presence of a screen protecting the face could reduce the amplitude of this response. This work aimed to investigate whether the learning of a posture intended to protect the head could modulate the HBR responses. Boxing athletes learn a defensive posture consisting of blocking with arms opponent’s blow towards the face. Two groups were recruited: 13 boxers and 13 people naïve to boxing. HBR response was recorded and elicited in three hand positions depending on the distance from the face. A suppression of HBR enhancement in the static position close to the face was observed in boxer group, contrary to the control group. Also, the higher years of practice in boxing, the higher suppression occurred. However, this suppression was not observed when boxers were asked to move the hand up-to/down-from the face. These findings might suggest that the sensorimotor experience related to a previously learnt protective posture can modify the HBR and thus shape the dimension of the DPPS.

## Introduction

The peripersonal space (PPS) is the space directly surrounding the body at grasping distance^1^. When the PPS contains obstacles or dangers from which we must defend its function becomes the body protection and for this reason it is defined defensive PPS (DPPS). Its modulation is fundamental when we interact with the surrounding environment^2,3^. DPPS can be investigated through the Hand Blink Reflex (HBR), a subcortical response at the brainstem level elicited by the electrical stimulation of the median nerve at the wrist and recorded from the orbicularis oculi muscles. HBR dramatically increases when the stimulated hand is statically positioned inside the PPS surrounding the face^4^. We recently tested HBR in dynamic conditions (i.e., when subjects performed upper limb voluntary movements towards and far from the face) and we found that through the integration of efferent and afferent signals during movement, the safety boundary around the body is continuously shaped by the predictive motor system. In particular, the intensity of responses increases when the hand moves towards the face bringing the threat near to the subject, but decreases when the hand moves away from the face^5^. This shows that in dynamic conditions HBR modulation depends not only by the actual position of the stimulated hand, but also by the final position where the hand is expected to be at the end of the movement. In line with this assumption a fine somatotopical and cognitive tuning of HBR has been reported by Sambo and colleagues^6^. In particular, they showed that when a thin wooden screen is placed between the participants’ face and their hand the HBR enhancement by hand-face proximity is suppressed. Thus, the screen reduces the extension of the DPPS, so that the hand is never inside the peripersonal space of the face, even in the “near” condition. All these findings indicate that both the static and the dynamic positions of the hand inside the DPPS of the face induce an HBR enhancement, but its amplitude is significantly influenced by the activity of motor, cognitive and associative cortical areas^4,6^. A strict connection between PPS and motor experience has been shown already in seminal papers on monkeys^7^ and in more recent papers with high technological methods (e.g. virtual reality^8^), it emerges the strict connection between PPS and motor experience^9^. This link seems to be particularly evident in the case of tool use. When subjects manipulate a tool to interact with the external world, like in the case of blind cane-user^10^, or simply use an object everyday, like in the case of a computer-mouse^11^, modifications in their PPS were observed. DPPS constitutes a protective interface to the external world and is known to be modulated by complex factors such as expectation of the risk^6^, personality traits^12^, presence of other people^13^. One might assume that also DPPS depends on previously acquired sensorimotor experiences that would trigger appropriate responses^14^, which in turn could result in the modulation of HBR. In other words, one could hypothesize that gaining a sensorimotor experience after an intensive and prolonged physical training, as occur in expert athletes, might have a long-term effects on peripersonal space representation.

In fighting sports, athletes learn to assume a specific posture to protect a part of body. In boxing, the danger for the athlete is represented by the punches of the opponent, especially those reaching the most delicate parts of the body such as the head and, in particular, the face. Therefore, to protect it, boxers are trained from the beginning of their practice to have a good boxing guard position. Although at a first glance the hands are inside the DPPS of the face, boxers are confident to use their hands as a shield to protect the face from an external threatening stimulus. Since this sport situation can be considered the ecological counterpart of the HBR experiment, we investigated whether this “shield effect” in boxers can shape the defensive peripersonal space surrounding the face.

A group of boxers with different years of practice were enrolled and compared with a group of 13 age-matched control subjects during a static HBR experiment hypothesizing that the expected HBR enhancement in the position close to the face would not be present in boxers, and that the years of practice could have a role in this modulation. Further, in order to understand whether the possible shaping of DPPS was due to the large experience of boxers in coping with dangerous stimuli when in a “guard” position (resembling the present static condition) or it was an unspecific phenomenon, not related to a particular body posture, boxers were involved also in a dynamic experiment. We recorded HBR when participants were asked to move their right forearm up towards the face (up-moving condition) or down far from the face (down-moving condition). Indeed, movements in different directions could allow us to investigate the response to a dangerous stimulus entering or leaving the DPPS.

## Materials and Methods

### Participants

Twenty-six participants, naive to the purpose of the experiment, were recruited for this study. The Boxer group (n = 13, 13 males, mean age ± SE = 28.27 ± 7.00 years) practiced for different amount of years: some of them participated to local and regional tournaments, but none of them competed at national level. They practiced boxing from 5 to 20 years.

The Control group was formed by volunteers who never practiced fighting sports (n = 13 males, mean age ± SE = 26.86 ± 4.29 years).

All the participants reported no previous history of neurological disorders or orthopedic problems for the right-dominant hand, as determined by the Edinburgh Handedness Inventory^15^.

Participants gave written informed consent before taking part in the study. The study has been approved by the local ethics committee (Comitato Etico Regionale Liguria, IRCCS Azienda Ospedaliera Universitaria San Martino – IST, Genova, Italy; P.R. 452REG2015) and was conducted in accordance with the Declaration of Helsinki.

### Experimental set up

The HBR response was elicited by administering transcutaneous electrical stimuli to the median nerve at the right wrist, using a surface bipolar electrode attached with a velcro strap and connected to a Digitimer constant current stimulator (DS7AH HV, Digitimer Ltd, UK). Stimulus intensity was adjusted to elicit in each participant clear HBR responses. None of the participants reported painful sensations. The stimulus duration was 200 μs and the inter-stimulus interval was about 30 s. In dynamic condition a twin-axis electronic goniometer (TSD130B, BIOPAC System, Inc.) connected to a BIOPAC MP100 system was used to measure and record the elbow angle during movement execution, allowing the automatic delivery of the electrical stimulation when the elbow angle corresponded to one of the three pre-determined stimulation positions.

EMG activity was recorded by means of two MP100 BIOPAC EMG channels from the orbicularis oculi muscles bilaterally, using two pairs of bipolar surface electrodes with the active electrode over the mid lower eyelid and the reference electrode laterally to the outer canthus. Signals were amplified and digitized at 1 kHz (BIOPAC MP100).

### Experimental Procedure

Participants were seated on a comfortable chair with their right elbow placed on a table, in a position allowing the right wrist to be in front of the ipsilateral eye while moving the forearm towards the face, but never touching it. The electrical stimulation was delivered while participant’s stimulated hand was located at three different positions relative to the face. In particular, when the elbow angle was 10° less than the maximal arm extension (far position, α1), the half of the difference between the angles of maximal arm extension and flexion (intermediate position, α2), and when the angle was 10° more than the maximal elbow flexion (near position, α3). Throughout the experiment participants were instructed to keep their gaze on a fixation point placed at 60 cm from the eyes.

**Figure 1:**
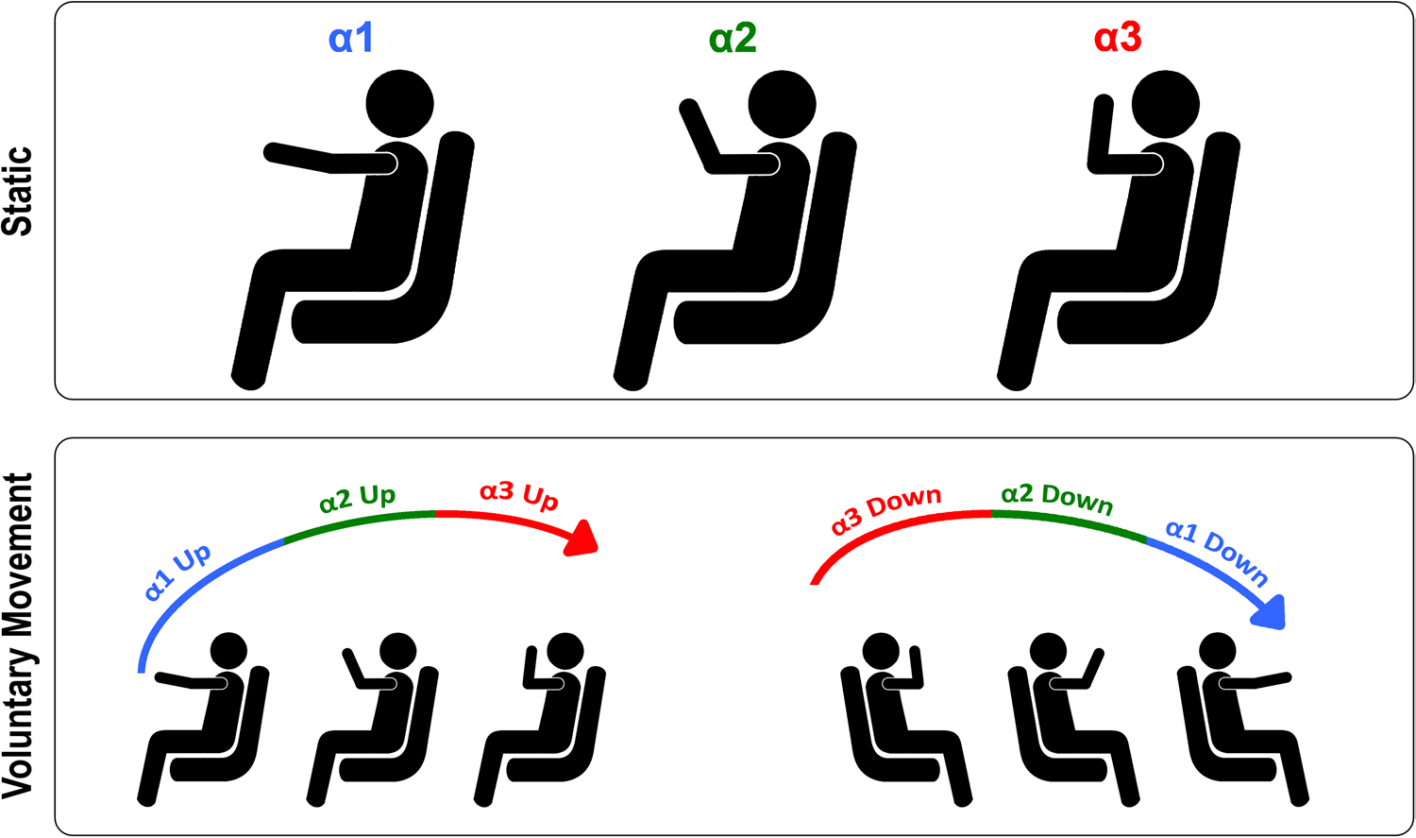
Experimental set up. Upper panel refers to static condition, during which subjects kept the arm and receive the stimulation in three target positions: positions (far, α1; intermediate, α2; near, α3). Lower panel refers to Voluntary movements condition, during which participants voluntarily performed either a flexion-extension or an extension-flexion movement of the elbow: while moving up towards (Up) or down far from (Down) the face they received an electrical stimulation in three pre-set positions (far, α1; intermediate, α2; near, α3).

The experimental paradigm consisted of two conditions performed in the same day: Static condition and Voluntary Movement condition (See Figure 1).

#### Static condition

At the beginning of each trial, participants had to assume with the right arm one of the three positions previously described, under experimenter’s instruction. After a randomly variable delay, they received the electrical stimulation, which was manually delivered by the experimenter. Static condition was performed before and after Voluntary Movement condition (3 stimulation positions, 4 repetitions and 2 times), for a total of 24 pseudo-random acquisitions, 8 for each hand position.

#### Voluntary Movement condition

Participants were asked to perform an elbow flexion-extension with the right arm, with the goniometer attached on it. The electrical stimulator was automatically triggered by the goniometer when the moving arm of the subject reached the target position previously set by the experimenter. Target positions were the angle values previously identified (α1, α2, α3), and the stimulation was delivered both during the elbow flexion (afterwards called Up-moving) or elbow extension (afterwards called Down-moving) movement, for a total of 48 trials (3 angles, 2 movement directions and 8 repetitions). To reduce participants’ expectancy, we introduced catch trials during which no stimulation was delivered. A minimum time of 30 sec was kept as inter-trial interval. During this interval the subjects were asked to keep the arm relaxed.

### Data processing and statistical analysis

A custom made MatLab software was used to process the EMG signals. EMG signals from each participant were filtered and rectified. HBR responses were averaged separately in each condition and for each participant. Trials with an abnormal EMG activity preceding the HBR responses were discarded from the analysis. The area under the curve (AUC, mV*ms) of each HBR average waveform was considered as outcome parameter. To compute AUC in each averaged EMG trace the software automatically analyzed a 130 ms-time interval from the stimulus onset that always contained the subject’s blink. The resulting curve was then integrated to compute AUC. In all experiments, data were averaged across ipsilateral and contralateral recording sides (right and left eyes).

In the Static condition, acquired AUC values were compared by mean of a repeated-measure ANOVA with POSITION (3 levels: α1, α2 and α3) as within-subject factor and GROUP (2 levels: Control group and Boxer group) as between-subject factor.

In Voluntary Movement condition, AUC data were analyzed by mean of RM-ANOVA, with POSITION (3 levels: α1, α2 and α3), and MOVEMENT DIRECTION (2 levels: Up-moving, Down-moving), as within-subject factors, and GROUP (2 levels: Control group and Boxer group) as between-subject factor. Newmann-Keuls post hoc analysis was used to interpret significant interactions.

Furthermore, for both Static and Voluntary Movement condition, we calculated for each subject of the Boxer Group the difference between the averaged AUC in the farthest and nearest positions. The values resulting from HBR_α3_-HBR_α1_ will be hereafter called ΔHBR. ΔHBR allowed to specifically evaluate the increasing of HBR when the hand was close to the face, inside the DPPS with respect to the farthest position. Pearson’s correlation was applied to assess any relationship between the ΔHBR and the years of practice of subjects.

### Significance statement

The defensive peripersonal space (DPPS) has a crucial role for survival, and its modulation is fundamental when we interact with the environment, as when we move our arms. Here we focused on a defensive response, the hand blink reflex (HBR), known to increase when a threatening stimulus is perceived as inside the DPPS of the face.

We found that the typical HBR enhancement was not present in a group of boxing athletes in the position they learnt as safe during their sport activity. Furthermore, the absence of HBR enhancement correlated with the years of experience subjects had in boxing.

All these findings suggest that previously acquired sensorimotor experiences can be generalized to other contexts and used to evaluate the threat of a stimulus in the definition of the DPPS.

## Results

Statistical analysis of the average amplitudes showed that the factor POSITION significantly affected the amplitude of the HBR responses (F_(2,48)_ = 19.09, p < 0.01). Post hoc test showed a significant increase of AUC values in α3 (17.64 ± 1.59 mV*ms) with respect to α1 (13.91 ± 1.11 mV*ms, p < 0.01) and α2 (13.11 ± 1.14 mV*ms, p < 0.01).

**Figure 2:**
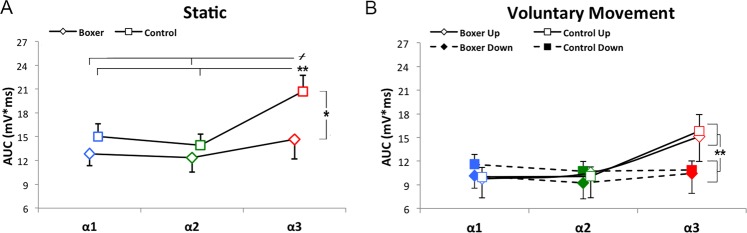
(**A**) Group-average HBR amplitudes (AUC,mV*ms) recorded when the arm was placed in the three stimulation positions: far (α1), intermediate (α2), and near (α3) in static condition. Squares indicate Control group, whilst diamonds indicate Boxer group. Error bar indicate standard error. ^**+**^p < 0.05 refers to the difference within the Control group, whilst *p < 0.05 and **p < 0.01 refers to the difference between groups. (**B**) Group-average HBR amplitudes (AUC,mV*ms) recorded in the up-moving (continuous lines) and down-moving (dashed lines) in the three stimulation positions: far (α1), intermediate (α2), and near (α3) in static condition. Squares indicate Control group, whilst diamonds indicate Boxer group. Error bar indicate standard error. **p < 0.01.

Furthermore, a significant interaction between POSITION and GROUP was found (F_(2,48)_ = 4.84, p = 0.012) and post hoc analysis revealed that the HBR responses of the Control group in α3 (20.64 ± 2.03 mV*ms) were significantly higher with respect to those acquired in the other positions (α1 = 14.95 ± 1.62 mV*ms, p < 0.01; α2 = 13.86 ± 1.42 mV*ms, p < 0.01) and in the other group (α1 = 12.86 ± 1.53 mV*ms, p < 0.05; α2 = 12.35 ± 1.81 mV*ms, p < 0.05; α3 = 14.65 ± 2.45 mV*ms, p < 0.05). Conversely, this effect was not present in the Boxer group, where AUC values in α3 did not significantly differ from those in α1 and α2 (Figs 1 and 2A).

In the Voluntary Movement condition, the results of the ANOVA on AUC averaged values showed significant main effects of MOVEMENT DIRECTION (F_(1,24)_ = 6.16, p = 0.02) and POSITION (F_(2,48)_ = 18.70, p < 0.01), and a significant interaction between these factors (F_(2,48)_ = 18.84, p < 0.01). Post hoc analysis showed that there was a significant increase of the HBR responses in α3 during the elbow flexion movement (Up-moving) with respect to all the other conditions (Up-moving α1: 9.87 ± 1.34 mV*ms; Up-moving α2: 10.31 ± 1.65 mV*ms; Down-moving α1: 10.39 ± 1.02 mV*ms; Down-moving α2: 9.97 ± 1.17 mV*ms; Down-moving α3: 10.66 ± 1.37 mV*ms. p always < 0.01) (Fig. 2B). No significant differences emerged between the two groups of subjects (F_(1,24)_ = 0.09, p = 0.76).

**Figure 3:**
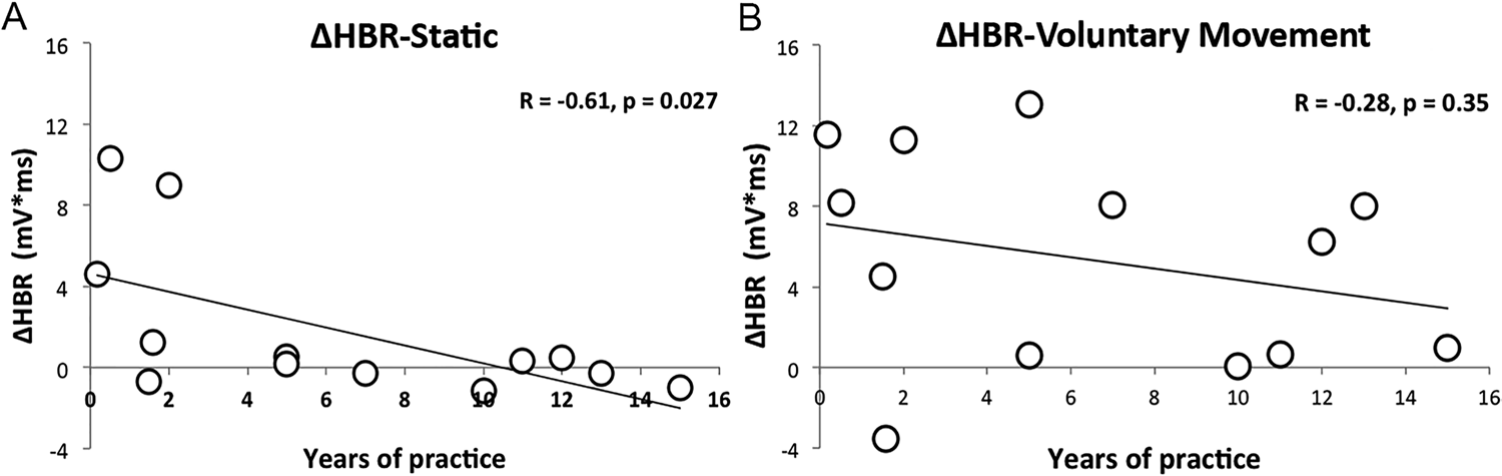
Pearson**’**s correlation between ΔHBR index and the boxers’ years of practice. ΔHBR indicates the intra-individual difference between subject-average response in α3 and α1 in Static condition (**A**) and in Voluntary Movement condition (**B**).

Figure 3 shows the relationship between the years of practice of Boxer group and the ΔHBR of their responses in Static and Voluntary Movement conditions.

A significant negative relationship was found in Static condition (*R* = −*0.61; p* = *0.027*); namely, the more years of practice they have, the less were the differences between the HBR responses in near and far position (Fig. 3A). On the contrary, no significant relationship was found between the years of practice in boxing and the ΔHBR of the Voluntary Movement condition (R = −0.28; p = 0.35; Fig. 3B).

## Discussion

The main finding of this study is the lack of modulation of HBR responses in expert boxers when the stimulated hand was statically placed near the face, effect that was present in the Control group. It is worth to note that the responses statistically differed between the two populations only when the stimulation was delivered in Static condition. Indeed, in the Voluntary Movement condition, the behaviour of boxer and control groups was basically the same, showing in both groups an increase of HBR responses when the stimulated hand in near position was moving towards the face.

Therefore the dissociation between static and dynamic condition in Boxer could be explained as a different boxers’ perception of the threat. We could assume that the near position mimics the guard posture, where boxers might perceive to be protected from dangers. The findings described by Sambo’s *et al*. study^6^ provide support to our explanation. In their study, when a wooden screen was placed in front of the participant’s face the amplitude of the HBR responses in near position did not increase^6^. Authors explained their result assuming that the screen shortened the boundaries of the DPPS of the face leading to perceive the hand outside it, even if it was in the same near position. We hypothesized that an analogue “screen effect” automatically manifested in boxers as consequence of their sport experience, because in guard position they use their hands as a shield to protect the face from an external threatening stimulus. The sensorimotor experience gained during years of practice in boxing might have contributed to the development of a contingent association between the own action (in this case, to parry from the opponent’s punch) and its outcome^16^. This learnt association might be transferred from the sports situation to a different but, in some respects, similar context, as in the case of the present experiment, during which a danger, namely the electrical stimulus, is present or is approaching the DPPS^17^.

The relation between motor experience and PPS was often noted when the manipulation of a tool was investigated. Indeed, it was shown that everyday tool use induced stably modifications to the users’ peripersonal space^10,11^. In sport context, a previous study of our group showed that the PPS of tennis players widened to include the personal racket (but not a common one), and this depended on the years of experience in their sport^18^. In the present study one might speculate that the upper limb of the boxers was a “tool” that defended the athlete from the external world. Boxers learn that when they assume their guard position they were secure and protected from the opponent; their motor experience changes the evaluation of the sensory events perceived at the level of the arm, whilst they have assumed a guard position. Instead, when boxers were still moving, the stimulus caught them unprepared. We could imagine that this condition mimics a situation during which an opponent’s hit reached the boxer when the guard was not completed. Indeed, it is possible that in Voluntary Movement conditions boxers did not feel to be in control of the parry action, and thus they were not confident in parry efficacy. Thus, not having the control over the effect let’s boxers to assess a greater risk when moving. At the same time, the movement proposed to the subjects during the Voluntary Movement conditions, namely a forearm flexion-extension, might not optimally represent boxers’ movement gestures when preparing for parry action. These would result in the increase of the perceived threat associated to the electrical stimulus, leading the HBR response to change in the same way as in the Control group, as observed in the present study.

It is worth to notice that HBR responses in Static condition were globally higher than the Voluntary Movement condition responses. This confirm the finding of our previous work^18^ in which we showed lower HBR values in all dynamic conditions. In that occasion, we hypothesized the existence of two distinct mechanisms underlying HBR in static and dynamic conditions. We also hypothesize an effect of sensory attenuation^19–22^, due to the fact that subjects triggered the stimulations with their own movement. If this was the case, boxers seemed to experience a greater control over threats originating from the external world with respect to those originating from themselves, likely because it represented an unfamiliar condition.

The main finding of this work is that a specific training to improve a body protection posture might stably modify the perceived threat of outside events, and consequently the HBR, namely a subcortical reflex response. This is further supported by the correlation between HBR modulation in static condition and the years of boxer’s experience; the higher the athletes’ experience the lower the HBR modulation, suggesting that athletes’ confidence on their “shield” increased the most experience they gained.

Taken together, these observations suggest that, as a result of sensorimotor experience related to a protective posture and gained during years of practice, the brain can shape the DPPS by evaluating the harm probability through the assessment of the ability to cope with possible dangerous stimuli. Furthermore, this corroborates the notion that cortical circuits exert a fine modulation of a subcortical reflex.
